# Comparative effects of 3,5-diiodo-L-thyronine and 3,5,3’-triiodo-L-thyronine on mitochondrial damage and cGAS/STING-driven inflammation in liver of hypothyroid rats

**DOI:** 10.3389/fendo.2024.1432819

**Published:** 2024-09-05

**Authors:** Antonia Giacco, Giuseppe Petito, Elena Silvestri, Nicla Scopigno, Michela Vigliotti, Giovanna Mercurio, Pieter de Lange, Assunta Lombardi, Maria Moreno, Fernando Goglia, Antonia Lanni, Rosalba Senese, Federica Cioffi

**Affiliations:** ^1^ Department of Science and Technologies, University of Sannio, Benevento, Italy; ^2^ Department of Environmental, Biological and Pharmaceutical Sciences and Technologies, University of Campania “L. Vanvitelli”, Caserta, Italy; ^3^ Department of Biology, University of Naples Federico II, Napoli, Italy

**Keywords:** mtDAMPs, oxidative stress, mitochondrial quality control, iodothyronines, hepatic dysfunction, hypothyroidism, inflammation

## Abstract

Maintaining a well-functioning mitochondrial network through the mitochondria quality control (MQC) mechanisms, including biogenesis, dynamics and mitophagy, is crucial for overall health. Mitochondrial dysfunction caused by oxidative stress and further exacerbated by impaired quality control can trigger inflammation through the release of the damage-associated molecular patterns (mtDAMPs). mtDAMPs act by stimulating the cyclic GMP-AMP synthase (cGAS) stimulator of interferon genes (STING) pathway. Recently, aberrant signalling of the cGAS-STING axis has been recognised to be closely associated with several sterile inflammatory diseases (e.g. non-alcoholic fatty liver disease, obesity). This may fit the pathophysiology of hypothyroidism, an endocrine disorder characterised by the reduction of thyroid hormone production associated with impaired metabolic fluxes, oxidative balance and inflammatory status. Both 3,5,3’-triiodo-L-tyronine (T3) and its derivative 3,5-diiodo-L-thyronine (3,5-T2), are known to mitigate processes targeting mitochondria, albeit the underlying mechanisms are not yet fully understood. Therefore, we used a chemically induced hypothyroidism rat model to investigate the effect of 3,5-T2 or T3 administration on inflammation-related factors (inflammatory cytokines, hepatic cGAS-STING pathway), oxidative stress, antioxidant defence enzymes, mitochondrial DNA (mtDNA) damage, release and repair, and the MQC system in the liver. Hypothyroid rats showed: i) increased oxidative stress, ii) accumulation of mtDNA damage, iii) high levels of circulating cytokines, iv) hepatic activation of cGAS-STING pathways and v) impairment of MQC mechanisms and autophagy. Both iodothyronines restored oxidative balance by enhancing antioxidant defence, preventing mtDNA damage through the activation of mtDNA repair mechanisms (OGG1, APE1, and POLγ) and promoting autophagy progression. Concerning MQC, both iodothyronines stimulated mitophagy and dynamics, with 3,5-T2 activating fusion and T3 modulating both fusion and fission processes. Moreover, only T3 enhanced mitochondrial biogenesis. Notably, 3,5-T2, but not T3, reversed the hypothyroidism-induced activation of the cGAS-STING inflammatory cascade. In addition, it is noteworthy that 3,5-T2 seems more effective than T3 in reducing circulating pro-inflammatory cytokines IL-6 and IL-1B and in stimulating the release of IL-10, a known anti-inflammatory cytokine. These findings reveal novel molecular mechanisms of hepatic signalling pathways involved in hypothyroidism, which could be targeted by natural iodothyronines, particularly 3,5-T2, paving the way for the development of new treatment strategies for inflammatory diseases.

## 1 Introduction

Thyroid hormones, including 3,5,3’-triiodo-L-tyronine (T3) and its derivative 3,5-diiodo-L-thyronine (3,5-T2), play crucial roles in the regulation of metabolic processes within the body (1, 2). 3,5-T2, though less powerful than T3, has gained considerable attention in recent years for its biological activity (3, 4). Like T3, it primarily exerts its effects by influencing energy metabolism (5, 6). It has been shown to enhance mitochondrial activity (7–9), leading to increased energy expenditure (10), and thermogenesis (11, 12). This effect is particularly interesting for its potential in promoting weight loss and addressing obesity-related conditions (13, 14).

The liver, a primary target organ for thyroid hormones, including 3,5-T2, is integral to metabolic regulation. Recent studies suggest that 3,5-T2 is able to affect hepatic lipid metabolism, leading to a reduction in fat accumulation in the liver, a finding of significant importance in addressing non-alcoholic fatty liver disease (NAFLD) (13, 15). In addition, studies have attributed antioxidant and anti-inflammatory properties to 3,5-T2 (16, 17), suggesting its protective role against oxidative stress and inflammation-induced liver damage, both pivotal in various liver diseases.

Liver hypothyroidism, resulting from thyroid hormone imbalance, can significantly disrupt lipid metabolism, impair energy homeostasis, and compromise liver health (18, 19). The exact mechanisms by which 3,5-T2 affects liver physiology in the context of hypothyroidism warrant further investigation, given the profound implications for metabolic regulation (20–24).

Energy homeostasis is intricately tied to mitochondrial function, as mitochondria are central to energy production and regulation in the body. Given their dynamic nature and susceptibility to stress, maintaining mitochondrial integrity is essential. Mitochondria quality control (MQC) mechanisms, including maintenance of mtDNA to protect against oxidative stress, mitochondrial biogenesis to generate new mitochondria, dynamics to regulate shape and distribution of mitochondria and mitophagy to remove damaged mitochondria, are crucial for optimal cellular function (for review see 25, 26). In addition, defective mitochondria can release mitochondrial damage-associated molecular patterns (mtDAMPs), molecules that can signal cellular stress or damage and trigger an inflammatory response (27, 28). mtDAMPs include various mitochondrial components such as mitochondrial DNA (mtDNA), ATP, and formyl peptides (29). When mitochondria are damaged or stressed, these components can be released into the cytoplasm or extracellular space, where they can be recognized by pattern recognition receptors on the cells. Cytosolic mtDNA can be detected by the cytosolic DNA sensor cyclic GMP-AMP synthase (cGAS), which then activates the interferon genes (STING) pathway, triggering inflammatory and immune responses (30, 31). It has been demonstrated that hypothyroidism can lead to a pro-inflammatory state (32). Chronic low-grade inflammation associated with hypothyroidism can exacerbate the development of atherosclerosis, NAFLD, and other inflammatory conditions (19, 33). Inflammatory cytokines are often elevated in hypothyroid patients, highlighting the significant interplay between hypothyroidism and systemic inflammation (34). Studying the association between hypothyroidism and mtDAMPs is of great importance, as it may provide new insights into the mechanism driving inflammation in the state of hypothyroidism.

This study primarily aims to investigate the effects of 3,5-T2 and T3 administered to hypothyroid rats on liver oxidative stress, modulation of hepatocyte MQC, including DNA repair, dynamics, biogenesis and mitophagy and, for the first time, their response to mtDAMPs activated pathways. Since the effects of hypothyroidism on such processes are not well understood, it is an additional and interesting goal to investigate how hypothyroidism affects these mitochondrial processes.

## 2 Materials and methods

### 2.1 Animals

Male Wistar rats (Rattus Norvegicus, 250–300 g, from Charles Rivers) were kept one per cage in a temperature-controlled room at 28°C (thermoneutrality for rats) under a 12:12 h dark/light cycle and provided with standard diet (Mucedola) and tap water ad libitum. All animal protocols were approved by the Committee on the Ethics of Animal Experiments of the University of Campania “L. Vanvitelli” (Italy) and the Italian Ministry of Health (permit number: 704/2016-PR of the 15 July 2016; project number: 83700.1 of the 3 May 2015).

The minimum sample size was calculated based on a G* Power Test that was performed using software obtained from the University of Dusseldorf: http://www.gpower.hhu.de/. The power was 0.90, the effect size (f) was 1.2249, and the α was 0.05. Rats were divided in 6 groups, each consisting of 6 animals as described below. “Eu” group consisted of euthyroid animals injected with vehicle. “Hypo” group consisted of rats made hypothyroid by daily intraperitoneal (ip) administration of propylthiouracil, PTU (1 mg/100 g body weight) together with a weekly ip injection of iopanoic acid, IOP (6 mg/100 g body weight), for 4 weeks as previously reported (11, 35, 36). The combined administration of PTU and IOP results in a severe hypothyroidism and a powerful inhibition of all three types of deiodinase enzymes (3). The “Hypo +T2” and “Hypo+T3” groups were treated like the Hypo group, but in the last week the first group received an additional daily injection of 3,5-T2 at the dose of 25µg/100 g body weight, while the second one received a daily injection of T3 at the dose of 15µg/100 g body weight (**Supplementary Figure S1**). Thus, the effects we observed are the individual contributions of the two iodothyronines. The treatment doses of the two iodothyronines were selected for their ability to induce clear effects on whole body metabolism and on the liver and skeletal muscle mitochondrial respiration rate, without inducing significant changes in the animals’ body weight (36, 37). At the end of the experimental period, rats were anesthetized (ip injection of chloral hydrate at the dose of 40 mg/100 body weight) and euthanized. Organs were collected, weighted, and frozen by liquid nitrogen and stored at −80°C for further processing. Blood was collected via the inferior cava vein in tubes and centrifuged at 5000 g for 5 min. Obtained serum was immediately frozen.

### 2.2 Serum hormones and metabolites detection

Serum total T4 (TT4), total T3 (TT3) levels were determined by specific ELISA tests from Dia.Metra s.r.l. (Perugia, Italy) according to the manufacturer’s instructions. On serum samples 8-hydroxy-2’-deoxyguanosine (8-OHdG), index of oxidative stress, was quantified using a DNA/RNA Oxidative Damage ELISA kit from Cayman Chemical Company (Ann Arbor, MI, USA). Finally, serum AST levels were measured by ELISA Kits from Abcam (Cambridge, UK).

### 2.3 Serum cytokines detection

Serum levels of interleukins IL-1B and IL-6, IL-10 and TNFα were performed using quantitative MILLIPLEX^®^ assays platform based on Luminex^®^ xMAP^®^, furnished from Prodotti Gianni (Milan, Italy).

### 2.4 Determination of H_2_O_2_ and MDA in liver samples

Liver endogenous H_2_O_2_ levels were measured by using the hydrogen peroxide colorimetric assay kit (Abcam), according to the manufacturer’s instructions. Values were normalised on total protein amount, quantified by Bradford methods (38).

Liver lipid peroxidation products [thiobarbituric acid reactive substances (TBARS) also known as malondialdehyde-equivalents (MDA-equivalents)]were measured by Lipid peroxidation kit from Sigma. Briefly, liver samples were homogenized on ice in MDA lysis buffer, containing BHT, incubated for 5 minutes at RT and then centrifuge at 13000g for 3 minutes. To form MDA-TBA adduct, solution containing TBA was added to sample and incubated at 95°for 60 minutes, and cooled on ice for 10 minutes. Samples were pipetted onto 96-well plate and absorbance measured at 532 nm. Data were analysed through a calibration curve, obtained by plotting standards of MDA solutions at concentration of 2, 4, 6, 8 and 10 mM and respective absorbances.

### 2.5 Genomic DNA isolation and quantitative polymerase chain reaction

Total DNA was extracted from liver samples (from 20 mg of frozen tissues) using the Genomic-tip 20/G kit (Qiagen, Valencia, CA, USA) according to the manufacturer’s instructions. Quality and quantity of extracted DNA was determined spectrophotometrically at 260 and 280 nm using NanoDrop One C (Thermo Fisher scientific, USA). QPCR was performed on 15 ng of liver DNA extracts as reported by Santos (39), with minor modifications already described by us (40). Primers sequences: mtDNA long fragment (13.4 Kbp) 5’-AAAATCCCCGCAAACAATGACCACCC-3’ (sense)/5’-GGCAATTAAGAGTGGGATGGAGCCAA-3’ (anti-sense); mtDNA short fragment (235 bp) 5’-CCTCCCATTCATTATCGCCGCCCTGC-3’ (sense)/5’-GTCTGGGTCTCCTAGTAGGTCTGGGAA-3’ (anti-sense). QPCR amplificates were measured using PicoGreen dye (Invitrogen, Milan, Italy) through fluorescence plate reader (Tecan, infinite pro 200, Austria). DNA damage was quantified by comparing the relative efficiency of amplification of the long mtDNA fragment normalized to the amplification of the small mtDNA fragment. Specifically, relative lesion frequencies per 10 Kbp DNA was calculated by applying the Poisson distribution according to the formula:


Lesion frequency=−ln Ad/Ao,

where Ad means amplification of treated samples, and Ao represents amplification of controls.

### 2.6 Quantification of mtDNA copy number by RT-qPCR

Mitochondrial DNA content, also known as mtDNA copy numbers, was assessed by amplifying through real-time quantitative PCR (RT-qPCR), mitochondrial cytochrome c oxidase subunit II (COII, mitochondrial-encoded gene) and β-actin (nuclear-encoded gene) genes on 50 ng of liver DNA extracts already described (40). The primer sequences used were: COII: 5’-TGAGCCATCCCTTCACTAGG-3’ (sense)/5’-TGAGCCGCAAATTTCAGAG-3’ (anti-sense); β-actin: 5’-CTGCTCTTTCCCAGATGAGG-3’ (sense)/5’-CCACAGCACTGTAGGGG TTT-3’ (anti-sense). The average Ct values of nuclear DNA and mtDNA were obtained for each sample, performed in triplicate. The mtDNA content was calculated using ΔCt = average Ct nuclear DNA—average Ct mtDNA and then by applying the formula mtDNA content = 2ˆ^(2ΔCt)^.

### 2.7 Isolation of DNA from cytosolic, mitochondrial and nuclear fractions and RT-qPCR

Liver samples (50 mg) were homogenized using Potter-Elvehjem homogenizer in fresh isolation buffer (220 mM mannitol, 70 mM sucrose, 20 mM Tris-HCl, 1 mM EDTA, 5 mM EGTA and 0.1% fatty acid-free BSA pH 7.4). The homogenate was centrifuged at 1000 g for 10 minutes. The resulting pellet was used for the preparation of the nuclear fraction. The supernatant was used to prepare the cytosolic and mitochondrial fractions. The cytosolic, nuclear and mitochondrial fractions were then obtained and enriched by differential centrifugations as described by Dias (41). DNA was isolated from subcellular fractions obtained using Trizol reagent (Thermo Fisher) according to the experimental protocol for DNA isolation. The quality and quantity of each DNA sample were determined using the NanoDrop One C (Thermo Fisher). For RT-qPCR, 50ng of DNA were incubated in a reaction volume of 25 µL containing iTAq Universal SYBR Green Supermix and appropriate primers at a final concentration of 6pM. Amplification was performed using Quant Studio 5 (Thermo Fisher). Results were normalized to nuclear β-actin expression levels and the cytosolic/mitochondrial ratio of mtDNA-encoded genes (COII) was determined (42). The following primers were used: COII: 5’-TGAGCCATCCCTTCACTAGG-3’ (sense)/5’-TGAGCCGCAAATTTCAGAG-3’ (anti-sense); β-actin: 5’-CTGCTCTTTCCCAGATGAGG-3’ (sense)/5’-CCACAGCACTGTAGGG TTT-3’ (anti-sense).

### 2.8 Total RNA isolation from liver and qRT-PCR

Total RNA liver was isolated using TRIzol reagent (Invitrogen) as previously described (43). 1 µg of RNA was used to synthesize cDNA strands, according to the Quanti Tect Reverse Transcription Kit instructions (Qiagen, Hilden, Germany). The qRT-PCR was carried out in appropriate volume of 20 µL containing cDNA samples (2 µL), 50 nM gene-specific primers, SensiFAST™ SYBR No-ROX Kit (Meridian Bioscience, USA). Gene expression levels were measured using standard cycle parameters on a QuantStudio 5 System (Thermo Fisher Scientific), calculated by the 2^−ΔΔCT^ method and normalized to the housekeeping gene β-actin. The following gene targets were evaluated: ape1, dio1, dio3, mct10, mct8, ogg1, pgc1α, polΥ, thrα, thrβ. PCR primers were designed by using the Primer 3 program (relative sequences were reported in **Supplementary Table S1**) and furnished by Eurofins Genomics (Ebersberg, Germany).

### 2.9 Liver protein extraction and western blotting

Liver Proteins extraction and quantification were performed as already reported (43). Briefly, rat liver tissue was homogenized in lysis buffer containing 20 mmol Tris-HCl (pH 7.5), 150 mmol NaCl, 1 mmol EDTA, 1 mmol EGTA, 2.5 mmol Na2H2P2O7, 1 mmol b-CH3H7O6PNa2, 1 mmol Na3VO4, 1 mmol PMSF 1 mg·mL−1 leupeptin, and 1% Triton X-100 (Sigma-Aldrich, St. Louis, MO, USA) using an Ultra Turrax homogenizer and then centrifuged at 15,000 *g* for 15 min at 4°C. Protein concentration was assayed in the resulting supernatants using Bio-Rad Protein Assay kit based on the Bradford methods (Bio-rad Laboratories, USA). Liver lysates containing 30 µg protein were used for western blot analysis. Primary antibodies used were: AMBRA1, APE1, ATGL16L1, ATG5, CATALASE, cGAS, DIO1, DIO3, GPX1, GPX4, IKBα, LC3IIb, MCT8, OGG1, OMA1, OPA1, P62, P65, PARKIN, PRDX3, PGC1**α**, P-IKB**α**, P-TBK1, P-ULK1^(757)^, PINK1, POLγ, SOD2, STING, TBK1, THRβ, TOM20, ULK1(reference numbers and manufacturers are listed in **Supplementary Table S2**). Membranes were incubated with horseradish-peroxidase anti-rabbit IgG or anti-mouse IgG secondary antibodies (Abcam), as appropriate. Proteins expression was detected by a chemiluminescence protein detection method using a commercially available kit (Millipore). Chemioluminescence accumulation signals were quantified on a Bio-Rad ChemiDoc™ XRS, using dedicated software (Imagelab, Bio-Rad Laboratories). Protein levels were normalized to β-actin.

### 2.10 Citrate synthase activity

Citrate synthase (CS) activity was assessed on whole liver homogenates using Citrate Synthase Assay Kit from Sigma, according to the manufacturer’s instructions. Liver fragments were homogenized (Potter-Elvehjem homogenizer) in isolation buffer (220 mM mannitol, 70 mM sucrose, 20 mM Tris-HCl, 1 mM EDTA, 5 mM EGTA, and 0.1% fatty acid-free BSA pH 7.4). Obtained homogenate was centrifuged at 500 g for 10 min at 4°C and 10 µg of protein extract for each sample were used for the assay. Reaction kinetic was monitored by reading Absorbance at 412 nm each 10 seconds, for 1,5 minutes.

### 2.11 Statistical analysis

Results are expressed as means ± SEM. For multiple comparisons, one-way ANOVA (*post hoc* test: Student-Newman-Keuls) was performed, while, Two-tailed, unpaired Student’s *t*-test was used for the comparison between two groups (Graph Pad Prism 5 software, GraphPad). Differences were considered statistically significant at *p*<0.05.

## 3 Results

### 3.1 Establishment of the hypothyroid phenotype: body weight gain, metabolic parameters and hepatic thyroid hormone signalling

Compared to euthyroid condition, Hypo rats showed significantly reduced body weight (BW) gain by 57% during the whole experimental period, concomitant to a reduced food intake (FI) by 17%, in accordance to previous work (36). This trend persisted in the hypothyroid rats receiving 3,5-T2 or T3 compared to the healthy group (Eu) at the end of the 4-weeks treatment. However, when we analysed only the last week of treatment, we found a differential effect of both iodothyronines on BW gain compared to Hypo. In absence of significant differences of FI between all the Hypo groups, T3 reduced BW gain, while 3,5-T2 caused a significant increase (-4,4 ± 2 g and +9,8 ± 1.3 g vs. Hypo, respectively) (**Table 1**).

**Table 1:** Metabolic phenotype of Eu, Hypo, Hypo+T2 and Hypo+T3 rats.

	Eu	Hypo	Hypo+T2	Hypo+T3
*Body Weight gain (BWg), g*	105 ± 6	45.5 ± 3*	44.5 ± 2*	45.7 ± 2*
*BWg during the last week, g*	18.9 ± 1,4	0.56 ± 0.45*	9.8 ± 1.3*^#^	-4.4 ± 2*^+^
*Food intake (FI), g*	488 ± 12	402 ± 7*	395 ± 12*	412 ± 6*
*FI during the last week, g*	130 ± 5	88 ± 5*	80 ± 7*	82 ± 5.2*
*Visceral adipose tissue/BW*	0.047 ± 0.003	0,041 ± 0.0014*	0.030 ± 0.002*^#^	0.031 ± 0.2*^#^
*Liver weight/BW*	0.029 ± 0.0012	0.027 ± 0.001	0.027 ± 0.0006	0.029 ± 0.0007

Data represent mean ± SEM of five independent measurements (n = 5). One-way Anova, Student-Newman-Keuls post test was performed, *p<0.05 vs Eu, ^#^p<0.05 vs Hypo, ^+^p<0.05 vs Hypo+T2.

When compared to the Eu group, Hypo showed a significant reduction of visceral white adipose tissue/BW ratio by 12,7%. Compared to Hypo, both 3,5-T2 and T3 further reduced it by 26% (**Table 1**). Liver weight/BW ratio did not change between all the experimental groups.

To test the hypothyroid phenotype and efficacy of iodothyronine treatment, total serum levels of T4 (TT4) and T3 (TT3) were measured. Both TT4 and TT3 were significantly reduced in the Hypo group (-86,1% and -76% vs. Eu, respectively). Compared to Hypo, administration of 3,5-T2 did not alter either TT4 or TT3 levels. On the other hand, Hypo+T3 rats showed significantly increased circulating TT3 levels after administration of exogenous T3, a condition that can be defined as a hyper-T3 state (+117% vs. Eu and + 825% vs. Hypo, **Table 2**).The maintenance of circulating levels of thyroid hormones and their action depend on gland secretion as well as their peripheral metabolism. Hepatic gene expression of the major thyroid hormone transporters (mct8 and mct10), iodothyronine deiodinases (dio1 and dio3) and nuclear receptors (thrα1 and thrβ1) were evaluated.

**Table 2:** Hormonal and biochemical parameters measured in serum of Eu, Hypo, Hypo+T2 and Hypo+T3.

	Eu	Hypo	Hypo+T2	Hypo+T3
*Serum TT4 nM*	57 ± 4	7.9 ± 2*	7.2 ± 0.9*	7.1 ± 1.1*
*Serum TT3 nM*	0.89 ± 0.01	0.20 ± 0.04*	0.19 ± 0.03*	1.85 ± 0.08*^#+^
*Serum AST, pg/mL*	5136 ± 353.6	7082 ± 852.6*	3948.5 ± 384.3^##^	8305 ± 283.9*^+^

Data represent mean ± SEM of five independent measurements (n = 5). One-way Anova, Student-Newman-Keuls post test was performed, *p<0.05 vs Eu, ^#^p<0.05 vs Hypo, ^##^p<0.01 vs Hypo, ^+^p<0.05 vs Hypo+T2.

**Figure 1:**
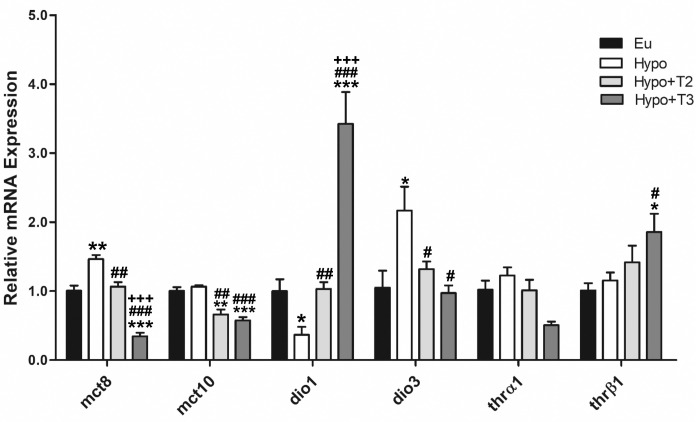
Hepatic gene expression of TH transporters (mct8 and mct10), iodothyronine deiodinases (dio1 and dio3) and TH receptors (thrα and thrβ) in Eu, Hypo, Hypo+T2 and Hypo+T3 groups. Values are presented as the means ± SEM from 5 rats in each group. One-way Anova, Student-Newman-Keuls post-test was performed, *p<0.05 vs Eu, **p<0,01 vs Eu, ***p<0,001 vs Eu; ^#^p<0.05 vs Hypo, ^##^p<0.01 vs Hypo, ^###^p<0,001 vs Hypo; ^+++^p<0.001 vs Hypo+T2. For Thrβ1 Student’s t-test was performed, *p<0.05 vs Eu and ^#^p<0.05 vs Hypo.

Hypo rats displayed a significantly increased expression level of mct8 transporter (+46% vs. Eu), while no changes were observed in mct10 expression. Both Hypo+T2 and Hypo+T3 rats showed reduced expression of mct8 (-23% and -76% vs. Hypo, respectively) and mct10 (-38% and -46% vs. Hypo, respectively) (**Figure 1**).

The expression levels of the major hepatic deiodinases, type 1 (dio1) and type 3 (dio3), were profoundly modulated under hypothyroid conditions, and even more so when iodothyronines were administered. Hypo rats showed a significantly reduced expression level of dio1 by 63%, paralleled by an increased expression of dio3 by 214%, compared to Eu. Dio1 expression was induced by 3,5-T2 and strongly increased by T3 (+186% and +836% vs. Hypo, respectively). Conversely, dio3 expression was significantly reduced by both iodothyronine treatment (-39% and -55% vs. Hypo, respectively) (**Figure 1**).

Compared to all experimental groups, thrβ expression was significantly enhanced in the Hypo+T3 rats (+85% vs. Eu, +60% vs. Hypo, +32% vs. Hypo+T2). There were no changes in the thrα expression across the experimental groups (**Figure 1**). In parallel, the protein expression of DIO1, DIO3, MCT8 and THRβ followed the same pattern as the gene expression (**Supplementary Figure S2**).

Liver gene expression analysis underpinned the efficacy of the chemical treatment used to induce hypothyroidism. Hypo rats exhibited decreased liver bioavailability of thyroid hormones as shown by decreased expression of dio1 and increased levels of dio3. Moreover, the increased expression of mct8 is likely a compensatory mechanism to increase tissue T3 levels while its circulating concentrations are low. This is consistent with the phenotype of chronic thyroid illness, in which patients with low serum T3 levels have the highest upregulation of MCT8 mRNA (44–46). 3,5-T2 stimulated dio1 mRNA as previously shown in mice (10) and decreased the expression of dio3. In Hypo+T3 rats, increased levels of circulating T3 were associated with decreased expression of dio3 and a strongly stimulated expression of both thrβ1 and dio1, this last being a thyroid hormone response element regulated T3-target gene (47).

Finally, the biochemical evaluation of serum showed that levels of aspartato aminotransferasi (AST), a circulating marker of liver damage, were significantly enhanced by 37% in Hypo compared to Eu rats. 3,5-T2, but not T3, was able to reduce AST levels by 44% compared to Hypo and normalize them to values comparable to those measured in Eu (**Table 2**).

### 3.2 3,5-T2 and T3 reduced oxidative stress and stimulated antioxidant defence in liver of hypothyroid rats

An imbalance between reactive oxygen species (ROS) and antioxidant enzymes leads to oxidative stress. Hypothyroidism-associated redox imbalance could be the result of increased free radicals production or reduced antioxidant defence capacity, or both.

**Figure 2:**
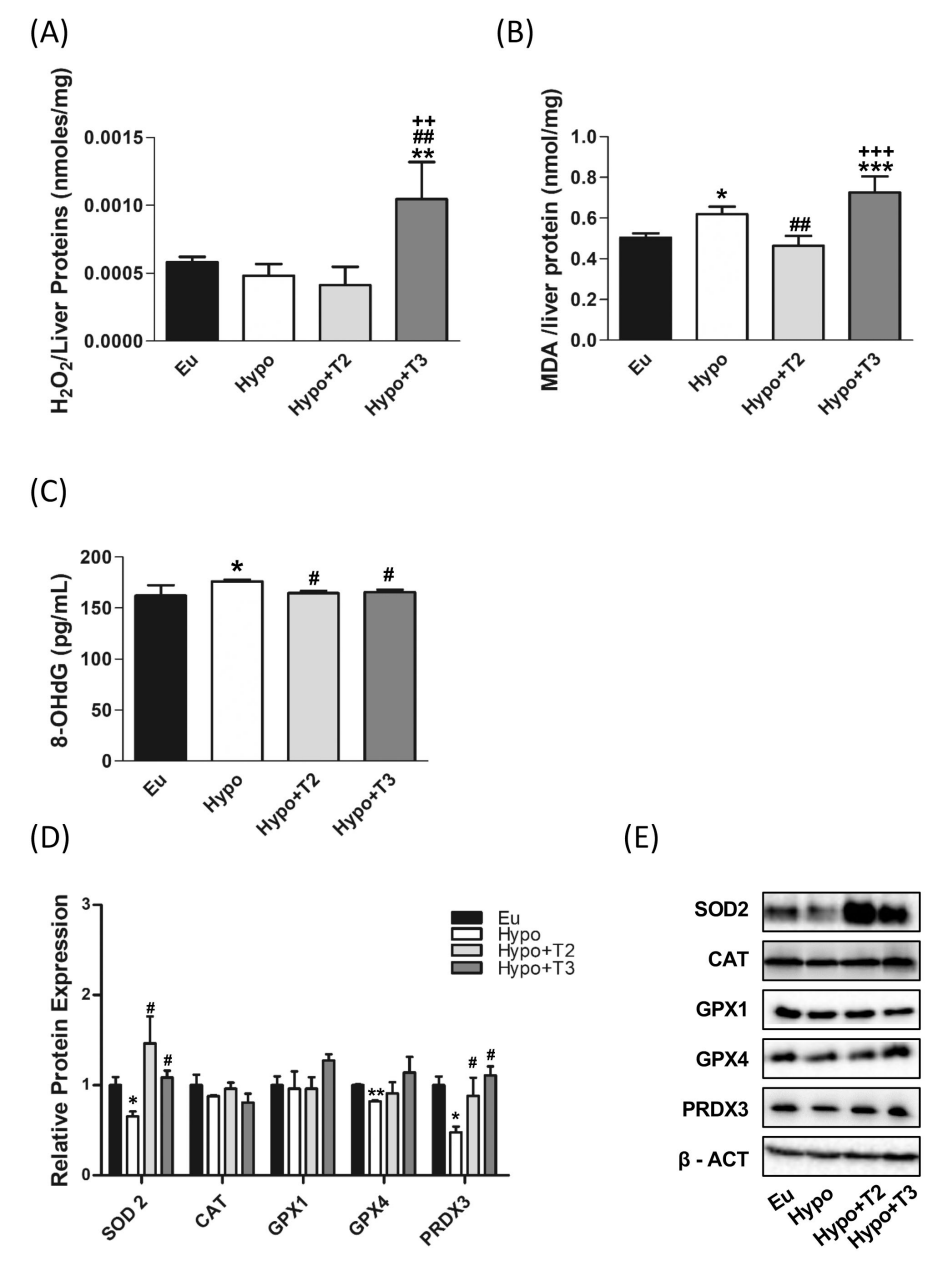
Oxidative stress and antioxidant defence enzymes in Eu, Hypo, Hypo+T2 and Hypo+T3 groups. **(A)** Hepatic H_2_O_2_ (nmol/proteins) level normalized on liver protein content; **(B)** hepatic MDA level normalized on liver protein content; **(C)** serum levels of 8-OHdG; **(D)** quantification of bands intensity of liver protein expression of SOD2, CAT, GPX1, GPX4 and PRDX3 and **(E)** representative western blot panel. Values are presented as the means ± SEM from 5 rats in each group, and normalised on value obtained from Eu set as 1. One-way Anova, Student-Newman-Keuls post-test was performed, *p<0.05 vs Eu, **p<0.01 vs Eu, ***p<0.001 vs Eu; ^#^p<0.05 vs Hypo, ^##^p<0.01 vs Hypo; ^++^p<0.01 vs Hypo+T2; ^+++^p<0.001 vs Hypo+T2. For SOD2 and GPX4 Student’s t-test was performed, *p<0.05 vs Eu; **p<0.01 vs Eu; ^#^p<0.05 vs Hypo.

To further determine how hypothyroidism affects the redox status in the liver, we measured tissue H_2_O_2_ concentrations, as index of ROS release, and MDA levels, as an index of lipid peroxidation. Hypothyroidism did not affect H_2_O_2_ levels, but significantly enhanced MDA levels by 22%, compared to the euthyroid state (**Figures 2A, B**). 3,5-T2 administration significantly reduced hepatic MDA level by 26% compared to the hypothyroid condition normalizing these parameter compared to the euthyroid state (**Figure 2B**). Hypo+T3 rats displayed statistically significant higher levels of H_2_O_2_ than all the other experimental groups (+79% vs. Eu, +116% vs. Hypo, and +153% vs. Hypo+T2) and increased levels of MDA when compared to Eu and Hypo+T2 (+44% vs Eu and +56% vs Hypo+T2) (**Figures 2A, B**).

To check liver antioxidant defence system in the model under study, expression levels of superoxide dismutase 2 (SOD2), catalase (CAT), glutathione peroxidase 1 and 4 (GPX1 and GPX4) and peroxiredoxin 3 (PRDX3) were measured. Western blot analysis showed a significant reduction in the expression of SOD2, GPX4 and PRDX3 in liver of hypothyroid compared to euthyroid animals (-34%, -17% and -48% vs. Eu, respectively). Catalase also showed a tendency to decrease in these animals, although it did not reach statistical significance. Compared to Hypo rats, Hypo+T2 and Hypo+T3 groups showed increased expression levels of SOD2 (+124% and +66% vs. Hypo, respectively) and PRDX3 (+84% and +132% vs. Hypo, respectively),whose levels resulted close to those measured for euthyroid rats (**Figures 2D, E**).

Finally, as a systemic marker of oxidative damage, serum levels of 8-OHdG, the major product of oxidative DNA damage, were measured. Hypo rats showed a significant accumulation of this oxidized base compared to euthyroid group (+10%). Both of the administered iodothyronines were able to significantly reduce 8-OHdG serum content under the hypothyroid state associated threshold restoring normal values (**Figure 2C**).

### 3.3 Both 3,5-T2 and T3 counteracted hypothyroidism-associated oxidative mtDNA damage, with only T3 stimulating mitochondrial biogenesis

Mitochondrial DNA is particularly prone to oxidative damage due to its proximity to the site where ROS are produced and its lack of protective histones. The QPCR assay for DNA damage is based on the principle that many kinds of DNA lesions can slow down or block the progression of DNA polymerase during PCR amplification (40). To measure the amplitude of mtDNA damage, the relative amplifications of long (13.4 kb) and short (235 bp) mtDNA fragments between all experimental groups were compared.

**Figure 3:**
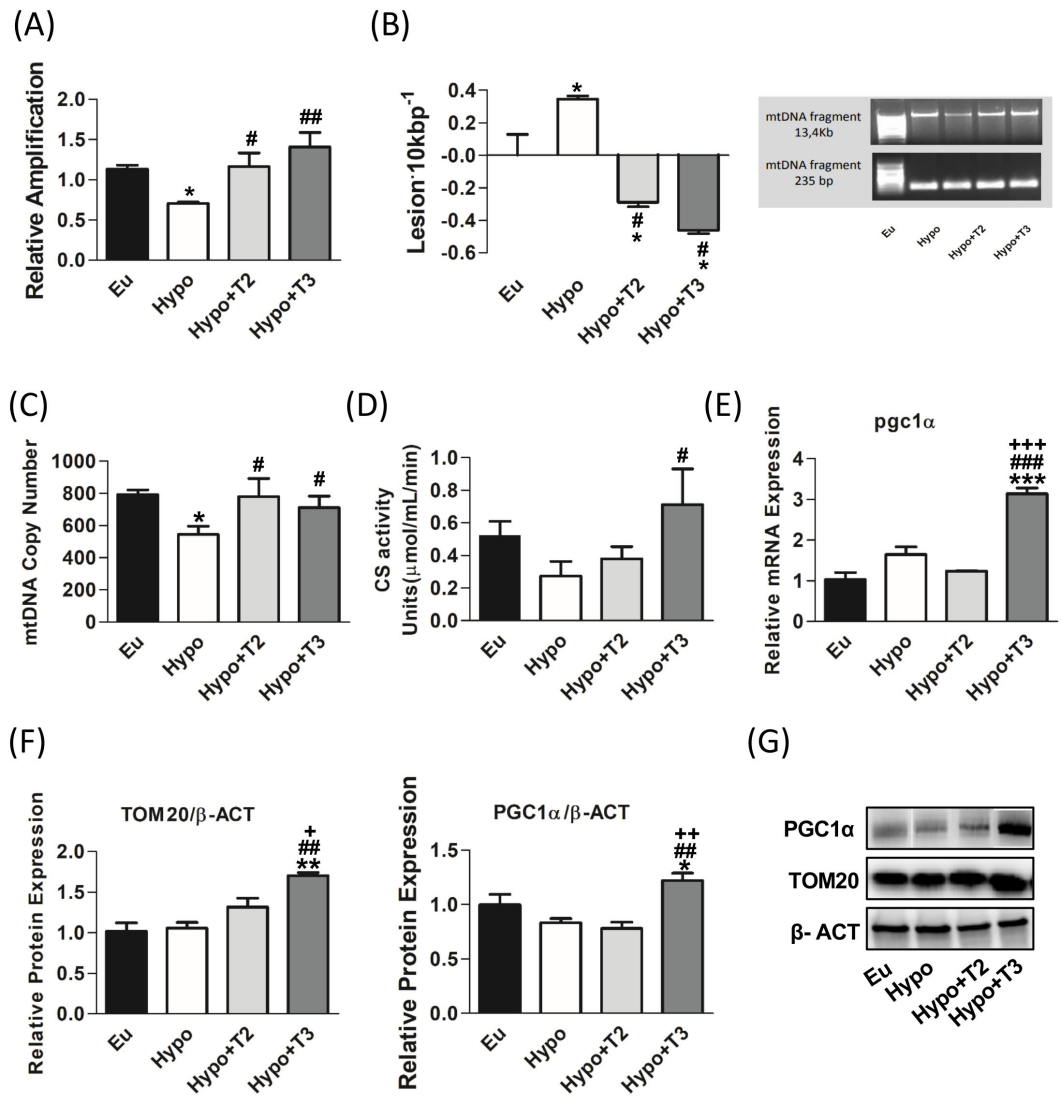
mtDNA damage and mitochondrial biogenesis in liver of Eu, Hypo, Hypo+T2 and Hypo+T3 groups. **(A)** mtDNA damage evaluated by amplifying long (13.4 kb) and short (235 bp) mtDNA fragments by QPCR; **(B)** frequency of mtDNA lesions per 10 kb per strand; **(C)** mtDNA copy number assessed by mtCOII amplification by Real Time PCR in 10 ng of genomic DNA; **(D)** CS activity on liver homogenates; **(E)** pgc1α gene expression; **(F)** quantification of bands intensity of liver protein expression of TOM20 and PGC1α and **(G)** representative western blot panel. Values are presented as the means ± SEM from 5 rats in each group. For E-G values were normalised on value obtained from Eu set as 1. One-way Anova, Student-Newman-Keuls post-test was performed, *p<0.05 vs Eu, **p<0.01 vs Eu; ***p<0.001 vs Eu; ^#^p<0.05 vs Hypo, ^##^p<0.01 vs Hypo, ^###^p<0.001 vs Hypo; ^+^p<0.05 vs Hypo+T2, ^++^p<0.01 vs Hypo+T2; ^+++^p<0.001 vs Hypo+T2.

As shown in **Figure 3A**, in hypothyroid rats, the relative amplification of the long mtDNA fragment on the short one was significantly reduced by about 37% compared to Eu group. 3,5-T2 and T3 both enhanced the relative amplification and counteracted mtDNA damage. Relative amplification was then converted to lesion frequency using the Poisson equation (**Figure 3B**). Liver mtDNA from Hypo rats contained significantly more mtDNA lesions compared to euthyroid rats (0.34 vs. 0.008 lesion·10 Kbp^−1^), while treatments with 3,5-T2 or T3 reduced the frequency of mtDNA lesions compared to Hypo (-0.28 and -0.46·10 Kbp^−1^ vs. Hypo, respectively) (**Figures 3A, B**).

Regarding markers of mitochondrial biogenesis, Hypo rats showed statistically significant lower levels of mtDNA copy number by 31,4% compared to euthyroid group (**Figure 3C**). In contrast 3,5-T2 increased the levels of mtDNA copy number by about 43% compared to the Hypo group. Neither the Hypo nor the 3,5-T2 group affected other mitochondrial biogenesis markers, such as CS activity, peroxisome proliferator-activated receptor gamma coactivator 1-alpha (PGC1α) and translocase of outer mitochondrial membrane 20 (TOM20) (**Figures 3D-G**). Conversely, compared to all the other experimental groups, T3 significantly enhanced CS activity and increased the expression of PGC1α, the master regulator of mitochondrial biogenesis, at both mRNA and protein levels (**Figures 3D-F**). In accordance, also the protein expression level of TOM20, a marker of mitochondrial mass, was significantly increased in Hypo+T3 compared to all the other groups (+67% vs. Eu, +61% vs. Hypo, and +29% vs. Hypo+T2) (**Figures 3F, G**).

### 3.4 Both 3,5-T2 and T3 stimulated the expression of markers of the mitochondrial base excision repair system in liver of hypothyroid rats

The mtBER system plays a central role in the correction mtDNA oxidative damage. Therefore, the presence of BER in mitochondria is crucial for maintaining the integrity of mtDNA and overall mitochondrial function. The enzymes involved in this system are: DNA glycosilase 1 (OGG1), apurinic/apyrimidinic endonuclease 1 (APE1), together with DNA polymerase gamma (POLγ). Both mRNA and protein expression levels of the above-mentioned markers were measured in all the experimental groups.

**Figure 4:**
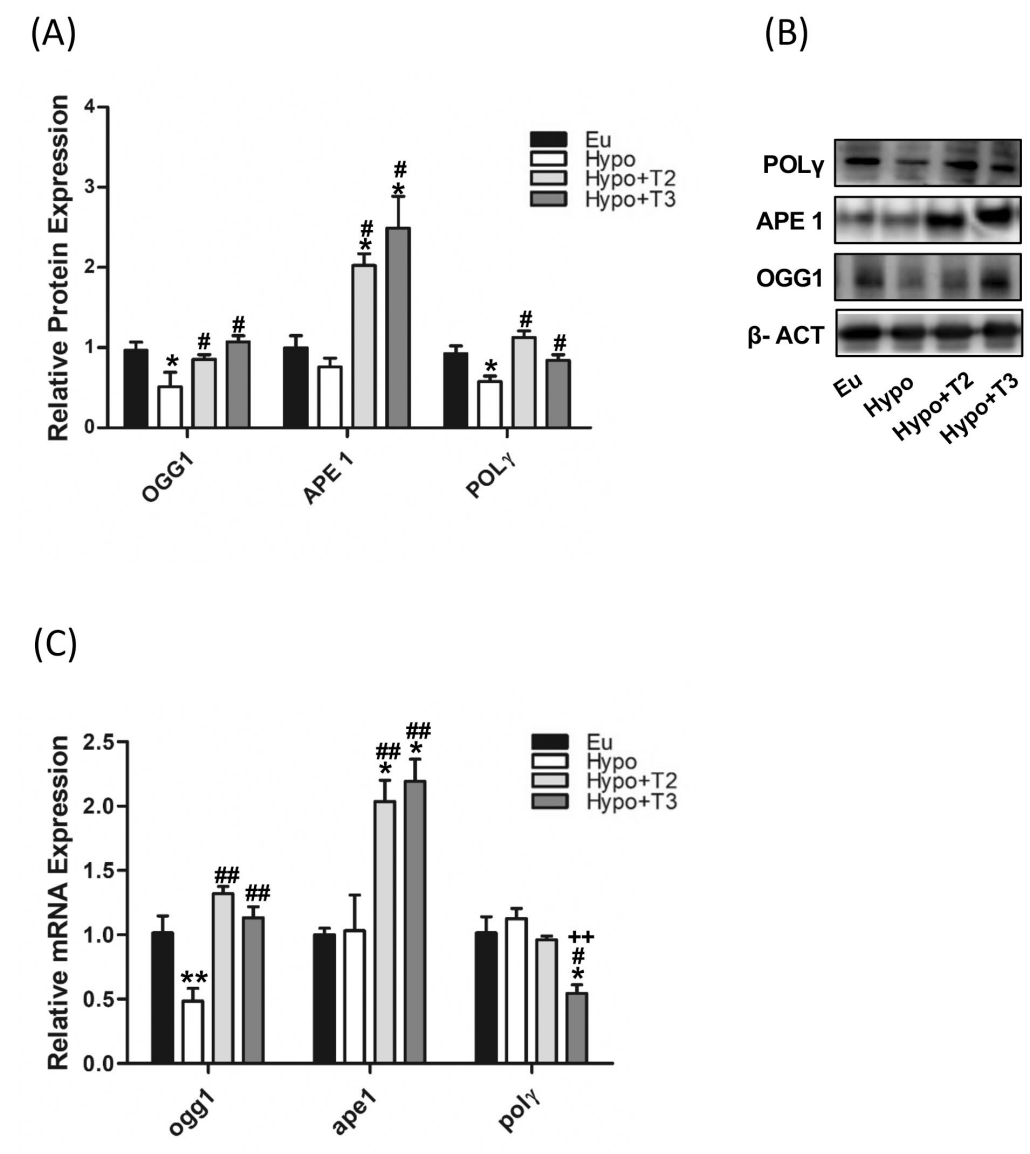
mtBER in liver of Eu, Hypo, Hypo+T2 and Hypo+T3 groups. **(A)** Quantification of bands intensity of protein expression of OGG1, APE1 and POLΥ **(B)** and representative western blot panel; **(C)** gene expression of ogg1, ape and polΥ. Values are presented as the means ± SEM from 5 rats in each group and were normalised on value obtained from Eu set as 1. One-way Anova, Student-Newman-Keuls post-test was performed, *p<0.05 vs Eu, **p<0.01 vs Eu; ^#^p<0.05 vs Hypo, ^##^p<0.01 vs Hypo; ++p<0.01 vs Hypo+T2.

As shown in **Figures 4A, B**, compared to euthyroid animals, Hypo rats showed significantly reduced protein expression levels of OGG1 and POLγ (-46% and -42% vs. Eu, respectively), paralleled by decreased transcriptional expression of OGG1 only (-52%) (**Figure 4C**). The expression of APE1 did not change under hypothyroid condition. Of note, administration of either 3,5-T2 or T3 increased the protein expression levels of OGG1 (+67% and +109% vs. Hypo, respectively), APE1 (+167% and +229% vs. Hypo, respectively), and POLγ (+89% and +41% vs. Hypo, respectively) with a parallel even more strongly increased mRNA expression of ogg1 (+173% and +143% vs. Hypo, respectively) and ape1 (+72% and +112% vs. Hypo, respectively) compared to Hypo rats (**Figures 4A-C**).

### 3.5 Hypothyroidism and iodothyronines modulated mitochondrial dynamics and autophagy/mitophagy

Together with mitochondrial biogenesis and mitophagy, mitochondrial dynamics is one of the central players in MQC. Thyroid hormones influence mitochondrial dynamics by modulating both fusion, in which two mitochondria are joined together, and fission, in which one is separated into two mitochondria, however the underlying mechanisms are not yet fully understood. The proteins optic atrophy 1 (OPA1) and OMA1 zinc metallopeptidase (OMA1) were selected as markers of mitochondrial fusion, while dynamin-related protein 1 (DRP1) of mitochondrial fission.

**Figure 5:**
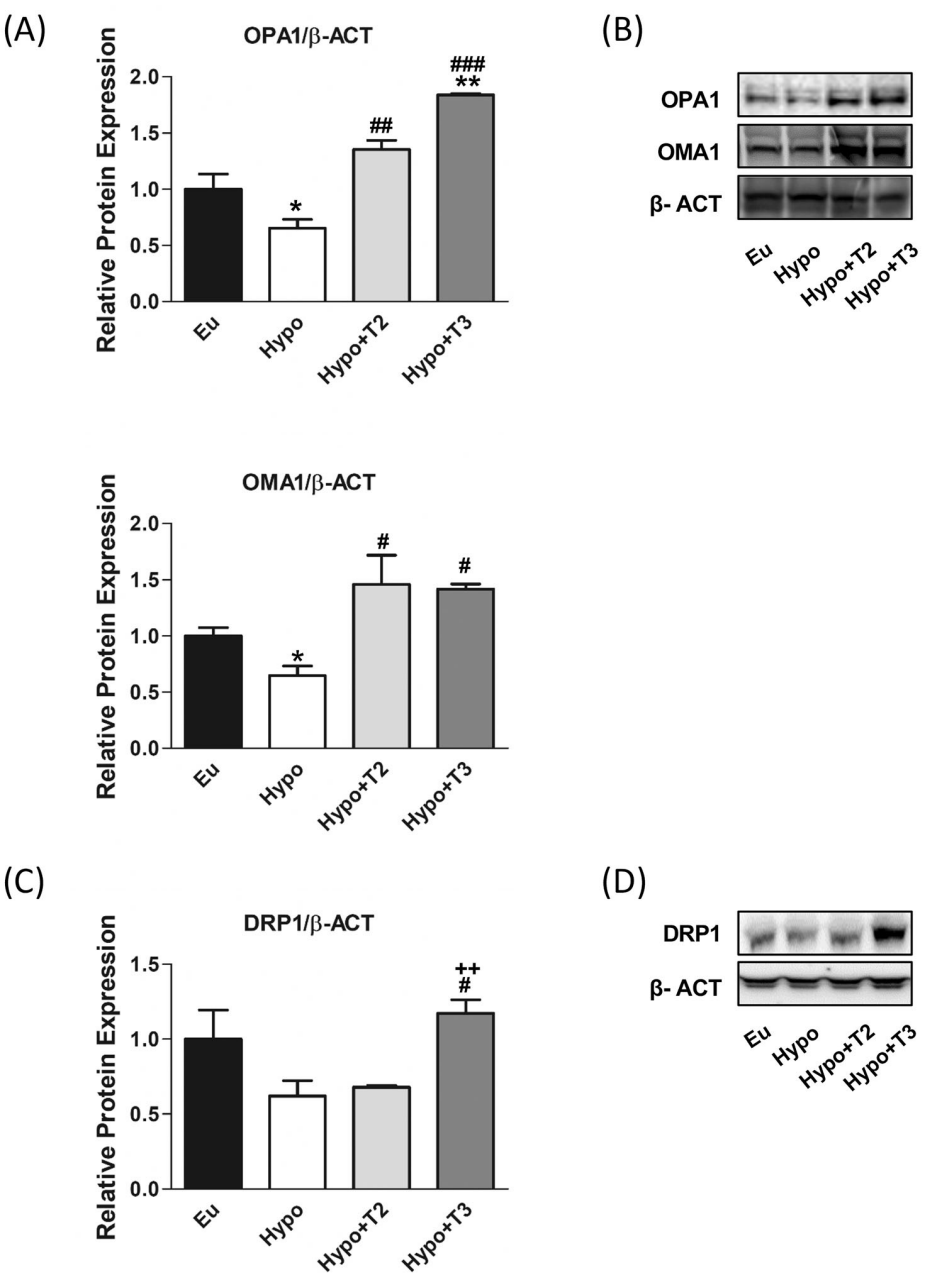
Mitochondrial dynamics, fusion and fission markers in liver of Eu, Hypo, Hypo+T2 and Hypo+T3 groups. **(A)** Quantification of bands intensity of liver protein expression of OPA1 and OMA1 **(B)** and representative western blot panel; **(C)** quantification of bands intensity of liver protein expression of DRP1 and **(D)** representative western blot panel. Values are presented as the means ± SEM from 5 rats in each group and were normalized on value obtained from Eu set as 1. One-way Anova, Student-Newman-Keuls post-test was performed *p<0.05 vs Eu; **p<0.01 vs Eu; ^#^p<0.05 vs Hypo, ^##^p<0.01 vs Hypo, ^###^p<0.001 vs Hypo. For DRP1 Student’s t-test was performed ^#^p<0.05 vs Hypo, ^++^p<0.01 vs Hypo+T2.

**Figures 5A, B** shows that the expression of OPA1 and OMA1 proteins was reduced by about 45% in hypothyroid rats compared to the euthyroid group, while the expression of DRP1 protein did not change (**Figures 5C, D**). Compared to Hypo, administration of 3,5-T2 or T3 significantly increased the expression of OMA1 and OPA1: (i) 3,5-T2 by 107% and 128%, respectively; (ii) T3 by 181% and 121%, respectively. Only administration of T3 significantly doubled the expression level of DRP1.

Autophagy that involves the delivery and degradation or recycling of cytoplasmic material at the lysosomes is important for removing dysfunctional components. A dysregulated and uncontrolled autophagy process elicits pathological responses consistent with hypothyroidism. Here we analysed the expression level of key proteins involved in the major steps of the autophagy process: activating molecule in beclin-1-regulated autophagy protein 1 (AMBRA1) and unc-51 like autophagy activating kinase 1 (ULK1), which triggers the formation of autophagosomes, autophagy-related 5 (ATG5) and autophagy-related 16 like 1 (ATG16L1), which together with autophagy-related 12 (ATG12) form a complex necessary for the progression of autophagy, microtubule-associated protein 1 light chain 3 beta (LC3B) and sequestosome 1 (P62), which are associated with the autophagosomal membranes in the final step that engulf cytoplasmic contents for subsequent degradation.

**Figure 6:**
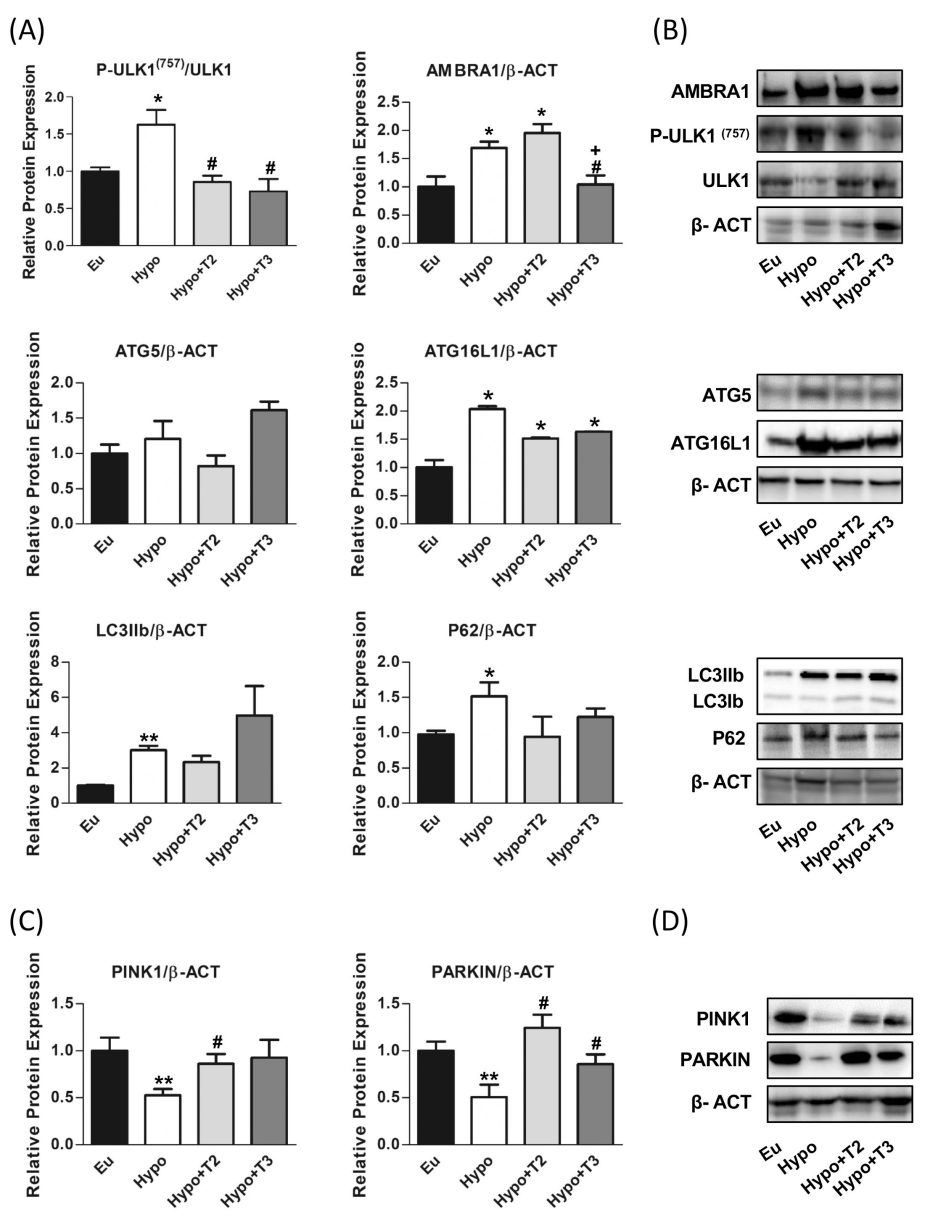
Autophagy and mitophagy in liver of Eu, Hypo, Hypo+T2 and Hypo+T3 groups. **(A)** Quantification of bands intensity of liver proteins expression involved in autophagy, P-ULK1^(757)^/ULK1, AMBRA1, ATG5, ATG16L1, LC3IIb, P62 **(B)** and representative western blot panel; **(C)** quantification of bands intensity of liver proteins expression involved in mitophagy, PINK1 and PARKIN and **(D)** representative western blot panel. Values are presented as the means ± SEM from 5 rats in each group and were normalised on value obtained from Eu set as 1. One-way Anova, Student-Newman-Keuls post-test was performed, *p<0.05 vs Eu; **p<0.01 vs Eu; ^#^p<0.05 vs Hypo; ^+^p<0.05 vs Hypo+T2. For P62 and PINK1 and LCIIb Student’s t-test was performed, *p<0.05 vs Eu, **p<0.01 vs Eu, ^#^p<0.05 vs Hypo.

As shown in **Figures 6A, B**, hypothyroid rats showed a significant upregulation of protein expression of AMBRA1, ATG16L1 and LC3IIB (+60%, +70% and +200% vs. Eu, respectively) indicating stimulation of the autophagic process. However, the parallel accumulation of P62 (+55% vs. Eu) indicates a blockage of the autophagic flux also supported by the increased inhibitory phosphorylation of P-ULK1 at Ser^(757)^ (+62% vs Eu). 3,5-T2 had no effect on the expression levels of ATG5, LC3IIb P62 proteins modulated under hypothyroidism. Compared to Hypo, T3 significantly reduced the expression levels of AMBRA1 by 38% and showed a trend towards stimulation of LC3II protein, although without statistical significance (p=0.22). Both 3,5-T2 and T3 normalized P62 expression levels to control condition, suggesting promotion of autophagy progression and significantly decreased P-ULK1 Ser^(757)^ phosphorylation. When autophagy is referred to as the mitochondria recycling process, it is known as mitophagy. Such a process can be non-selective or mediated by specific protein interactions, PTEN-induced kinase 1 (PINK1) and E3 ubiquitin ligase (PARKIN).

Hypo rats showed a halving of PINK1 and PARKIN expression compared to the Eu control group. PARKIN expression levels were significantly induced in Hypo+T2 and Hypo+T3 compared to the Hypo group (+63% and +75%, vs. Hypo, respectively), while PINK1 expression although increased by both iodothyronine treatment, reached statistically significance only in Hypo+T2 rats (+145% vs. Hypo) (**Figures 6C, D**).

### 3.6 Hypothyroidism and iodothyronines oppositely regulated cGAS-STING pathway in liver

**Figure 7:**
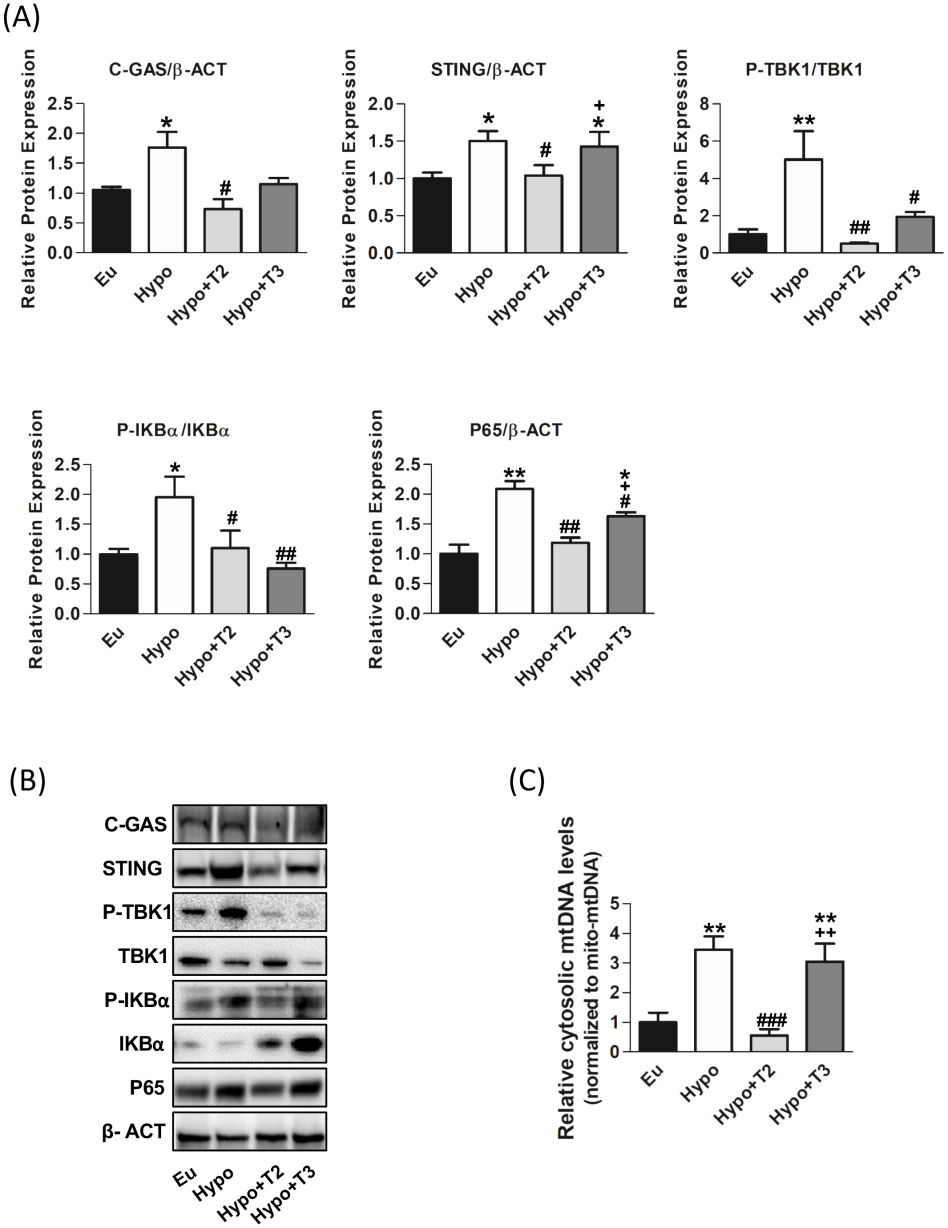
mtDAMPs inflammatory pathway in the liver of the Eu, Hypo, Hypo+T2 and Hypo+T3 groups. **(A)** Quantification of bands intensity of liver proteins expression involved in inflammation, cGAS, STING, P-TBK1/TBK1, P-IKBα, IKBα, P65 and **(B)** representative western blot panel; **(C)** RT-qPCR measurement of liver cytosolic mtDNA (mtCOII), normalized to mtDNA content in the mitochondrial fraction. Values are presented as the means ± SEM from 5 rats in each group and were normalised on value obtained from Eu set as 1. One-way Anova, Student-Newman-Keuls post-test was performed, *p<0.05 vs Eu, **p<0.01 vs Eu; ^#^p<0.05 vs Hypo, ^##^p<0.01 vs Hypo; ^###^p<0.001 vs Hypo; ^+^p<0.05 vs Hypo+T2; ^++^p<0.01 vs Hypo+T2.

The pro-inflammatory properties of various mitochondrial molecules once they leave the mitochondrial compartment have been extensively studied in recent years. These molecules namely mtDAMPs may serve as an important pro-inflammatory triggers in hypothyroidism. Among the DAMPs, mtDNA released in the cytosol activates the cGAS-STING-TBK1 cascade. Thus, we measured its content in the cytosol fraction. Hypo rats showed significant increased cytosolic mtDNA levels by 200% compared to Eu (**Figure 7C**).

As shown in **Figures 7A, B**, the expression of cGAS and STING proteins was significantly increased in Hypo rats by 64% and 46%, respectively, compared to Eu rats. Upon activation, cGAS/STING activate tank-binding kinase 1 (TBK1) through its phosphorylation. A significant upregulation of TBK1 phosphorylation by 500% compared to the Eu group was observed.

To confirm the pro-inflammatory status of liver of Hypo rats, activation of IkBα (through its phosphorylation) and the accumulation of p65, its active subunit, were measured. Hypothyroid rats showed a significant increase of P-IKBα/IKBα ratio and P65 protein levels (+95% and +109 vs. Eu, respectively).

Administration of 3,5-T2 to hypothyroid rats significantly reduced liver cytosolic mtDNA levels (-81% vs Hypo), the protein levels of cGAS and STING (-52% and -30% vs. Hypo, respectively). On the contrary, T3 was did not affect both cytosolic mtDNA levels and cGAS and STING protein levels compared to Hypo. Both 3,5-T2 and T3 decreased TBK1 (-90% and -60% vs. Hypo, respectively) and IKBα phosphorylations (-43% and -61% vs. Hypo, respectively) as well as p65 protein accumulation (-43% and 21% vs. Hypo, respectively) (**Figures 7A, B**).

### 3.7 3,5-T2 but not T3 restored the balance between pro and anti-inflammatory cytokines under hypothyroid condition

**Figure 8:**
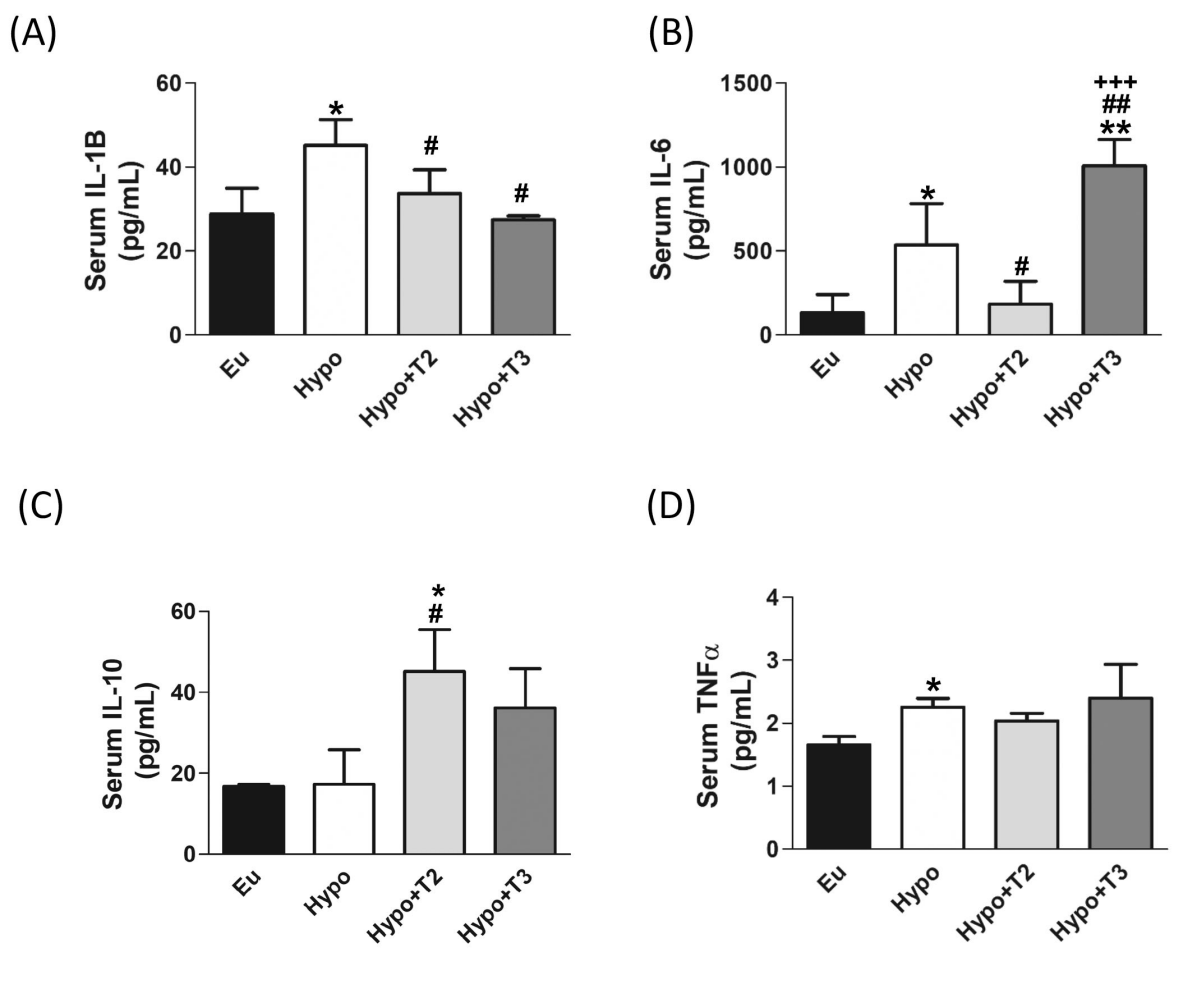
Circulating pro/anti-inflammatory cytokines in serum of the Eu, Hypo, Hypo+T2 and Hypo+T3 groups. Circulating levels of **(A)** IL-1B **(B)** IL-6 **(C)** IL-10 and **(D)** TNFα. Values are presented as the means ± SEM from 5 rats in each group and were expressed as pg/mL. One-way Anova, Student-Newman-Keuls post test was performed, *p<0.05 vs Eu, **p<0.01 vs Eu; ^#^p<0.05 vs Hypo, ^##^p<0.01 vs Hypo; ^+++^p<0.001 vs Hypo+T2. For TNFα Student’s t-test was performed *p<0.05 vs Eu.

Finally, to evaluate the systemic inflammatory status, pro-inflammatory (IL-6, IL-1B and TNFα) and anti-inflammatory (IL-10) cytokines were determined in the serum of all experimental groups using Milliplex technologies and the data are presented in **Figures 8A-D**. Compared to euthyroid rats, the Hypo group showed significantly increased levels of IL-1B, IL-6, and TNFα (by 56%, 300%, and 36% vs. Eu, respectively). Compared to the Hypo group, administration of 3,5-T2 significantly reduced circulating levels of IL-1B by 25% and of IL-6 by 65% (**Figures 8A, B**). On the other hand, 3,5-T2 treatment increased the serum levels of the anti-inflammatory IL-10 (+167% vs. Hypo) (**Figure 8C**). Hypo+T3 rats showed reduced serum level of IL-1B by about 40% compared to Hypo and an increased level of IL-6, the latter statistically significant compared to all the other experimental groups (+660% vs. Eu, +87% vs. Hypo, and + 450% vs. Hypo+T2) (**Figures 8A, B**).

## 4 Discussion

In our study, we investigated the effects of 3,5-T2 and T3 on mitochondrial integrity, including oxidative damage, MQC mechanisms and cGAS-STING pathway in the liver of hypothyroid rats. Thyroid disorders like hypothyroidism can affect various aspects of liver health and metabolism. Previous studies have shown a correlation between thyroid disorders and heightened liver enzymes such as AST and ALT. Elevated levels of these enzymes in the bloodstream signal liver dysfunction stemming from hepatocellular injury (48–53). Our results demonstrate that rats with hypothyroidism, with and without T3 treatment, had elevated serum AST levels, indicating liver damage or dysfunction. Nevertheless, the administration of 3,5-T2 exhibited a hepatoprotective effect, as evidenced by the reduction in serum AST levels.

The elevated AST levels could be a consequence of oxidative stress damage to liver cells. Our findings are consistent with previous studies that reported increased lipid peroxidation in the liver and increased serum 8-OHdG levels, both indicative of increased oxidative damage to the liver in hypothyroidism (52, 54–58 *).* This is associated with a reduced expression of the mitochondrial antioxidant enzymes SOD2, GPX4 and PRDX3, suggesting of impaired liver antioxidant defences. Additionally, catalase, another important antioxidant enzyme, also showed a tendency to decrease in hypothyroid animals, further suggesting impaired antioxidant defences. The mechanism underlying increased oxidative stress in hypothyroidism is indeed a topic of ongoing debate (59, 60). One proposed explanation suggests that a deficient antioxidant defence system might contribute. In accordance with our results, the hypothesis states that the antioxidant defence system in hypothyroidism is insufficient to neutralise the increased production of free radicals, thus resulting in oxidative stress (61–63).

In the Hypo+T2 group, we observed a decrease in liver MDA content and serum 8-OHdG concentration, alongside an increase in the expression of SOD2 and PRDX3 in liver protein, suggesting a protective role of 3,5-T2 against oxidative stress in the liver. Our findings are in line with previous studies on the effects of 3,5-T2 on oxidative stress affecting redox balance in liver (64, 65). Mollica et al. (64) found that the administration of 3,5-T2 decreased hepatic mitochondrial oxidative stress, as evidenced by a significant decrease in hydrogen peroxide (H_2_O_2_) levels.

T3 treatment can affect the redox status in the liver, leading to changes in oxidative stress markers and antioxidant defence mechanisms (66, 67). Our results, consistent with existing literature, showed an increase in H_2_O_2_ and MDA levels in the liver of Hypo+T3 rats. One explanation may lie in the supraphysiologic T3 dose we used in this and in a previous study (36) to treat hypothyroid rats, which has been shown to significantly stimulate mitochondrial oxidative metabolism. However, the increased expression of the antioxidant enzymes SOD2 and PRDX3 in the liver suggests that T3 treatment reinforces the antioxidant defence system, thereby mitigating the oxidative damage caused by H_2_O_2_ and lipid peroxidation. Interestingly, despite the local increase in ROS production in the liver, the heightened activity of these antioxidant enzymes may lead to a decrease in systemic markers of oxidative stress, such as 8-OHdG. Our data suggest that both 3,5-T2 and T3 positively influence the removal of mtDNA damage, consequently leading to a reduction in serum 8-OHdG levels. The clearance of mtDNA damage involves cellular processes aimed at either repairing or eliminating damaged mtDNA molecules. Cells utilize several mechanisms, including DNA repair pathways and quality control systems, to maintain the integrity of their mtDNA (68, 69).

Under hypothyroid conditions, we observed a decrease in the expression of two proteins involved in both upstream and downstream of mtBER, such as OGG1 and POLγ. In particular, the decrease in OGG1 and POLγ proteins suggests a possible impairment in the maintenance of mitochondrial DNA integrity in hypothyroidism. This could contribute to the accumulation of oxidative DNA damage in the mitochondria, which is likely a consequence of the oxidative stress observed in hypothyroid rats. This stress can lead to significant DNA damage in the mitochondrial genome, including the formation of oxidised bases and strand breaks. We reported an increase in the frequency of mtDNA lesions and a decrease in mtDNA copy number in the livers of hypothyroid rats. These observations are indicative of disturbances in mitochondrial biogenesis and mtDNA replication processes, likely related to decreased expression of POLγ. Administration of 3,5-T2 and T3 to hypothyroid rats significantly increases the expression levels of OGG1, APE1 and POLγ proteins, which all play a key role in mtBER mechanisms. This enhancement potentially restores mitochondrial DNA repair capacity and mitigates the deleterious effects of oxidative stress on mitochondrial integrity.

In a previous study we obtained parallel results showing that administration of 3,5-T2 and T3 to euthyroid rats can similarly reduce, in the liver, oxidative damage to mtDNA. At that time, we hypothesized that although similar effects on mtDNA lesions, 3,5-T2 and T3 acts through different mechanisms, 3,5-T2 primarily repairing lesions, T3 stimulating mitochondrial biogenesis, thus producing a mitochondrial population enriched with new, less damaged mitochondria (70). Consistently, in the present study, we observed in the liver of Hypo+T3 rats increased mtDNA copy number, CS activity and PGC1α and TOM20 expression, all key markers of mitochondrial mass (71–73). In addition, we observed that administration of 3,5-T2 increased mtDNA copy number and restored it to normal in euthyroid animals without affecting PGC1α activation. The mtDNA replication machinery could act as a mediator for this effect, as suggested by the increase of POLγ expression (74, 75).

Other mechanisms involved in the maintenance of mitochondrial integrity include mitochondrial fusion, fission and mitophagy as part of the MQC system. Mitochondrial fusion facilitates the exchange of contents between damaged and healthy mitochondria, while fission segregates damaged portions of mitochondria for degradation through autophagy, particularly mitophagy, which selectively targets damaged mitochondria for lysosomal degradation. Data obtained in the present study show that hypothyroidism significantly alters mitochondrial dynamics, leading to a decrease in OPA and OMA1 levels in the liver. The expression of DRP1 remained unchanged, suggesting intact mitochondrial fission. However, there are few studies investigating these processes specifically in the context of hypothyroidism (76). As OPA1 and OMA1 are critical for the maintenance of mitochondrial integrity and function, their decline suggests an impaired mitochondrial fusion process in hypothyroidism, likely leading to mitochondrial dysfunction and impaired cellular energy production. Indeed, OPA1 deficiency exacerbates the formation of respiratory chain supercomplexes (RCS), leading to reduced electron transport chain (ETC) activity and oxidative phosphorylation (77). In addition, numerous studies have reported that hypothyroidism leads to decreased mitochondrial respiration, further supporting the effects of thyroid hormone levels on mitochondrial function (12, 14, 36, 78–82).

On the other hand, the livers of hypothyroid rats treated with either 3,5-T2 or T3 exhibit increased mitochondrial fusion, as evidenced by increased OPA1 and OMA1 levels, possibly counteracting the deleterious effects of hypothyroidism on mitochondrial dynamics. To date, there are few studies directly linking thyroid hormone administration to fusion and fission processes (83, 84). Recent results showing the modulatory effect of 3,5-T2 on mitochondrial dynamics in skeletal muscle of high-fat diet (HFD)-fed rats emphasise its potential role in attenuating mitochondrial dysfunction (36). Overexpression of OPA1 and OMA1 in the liver has been associated with maintenance of mitochondrial homeostasis and function (85–88). In addition, when T3 is administered, the cell attempts to maintain mitochondrial quality and function even through the interplay of DRP1 overexpression and increased mitochondrial biogenesis. By promoting mitochondrial fission, T3 contributes to the formation of smaller, more dynamic mitochondria and the clearance of damaged mitochondria (89, 90) while increasing mitochondrial biogenesis to replenish the mitochondrial pool with new, functional organelles (72, 91). This is consistent with our previous data, according to which T3 increases mitochondrial oxidative capacity by increasing the abundance of respiratory chain components and favouring the organization of the respiratory chain complex into supercomplexes (36).

Mitophagy, a selective form of autophagy responsible for degrading damaged mitochondria, is crucial for maintaining a healthy mitochondrial population. In the liver of hypothyroid rats, we observed an upregulation of AMBRA1, ATG16L1 and LC3IIB proteins, which initiate the autophagy process. However, the observed accumulation of P62, a protein typically degraded by lysosome during autophagy induction, suggests a blockage and impairment of downstream processes of autophagic flux (92–94). This is supported by the increased phosphorylation of serine 757 inhibiting autophagy through the mechanistic target of rapamycin (mTOR) (95) in hypothyroidism. In addition, reduced expression of PINK1 and PARKIN indicates a potential impairment of mitophagy, possibly leading to an accumulation of damaged mitochondria, which could disrupt cell function (96, 97).

Several studies have investigated the role of T3 and 3,5-T2 in activating various forms of autophagy in the liver to maintain cellular quality control and regulate energy metabolism (98–101). In our study, administration of 3,5-T2 to hypothyroid rats promotes mitophagy, as evidenced by increased levels of PINK1 and PARKIN proteins in the liver. This suggests that administration of 3,5-T2 promotes MQC mechanisms in the liver. Furthermore, the selective increase in PARKIN levels in the liver following T3 administration indicates a specific enhancement of PARKIN-mediated mitophagy. Consistent with the induction of mitophagy by iodothyronines, both 3,5-T2 and T3 reduced the inactivating phosphorylation level of ULK1 at Ser^(757)^ (102).

In the context of hypothyroidism, our research has shown a significant impairment of cellular processes such as autophagy and mitophagy. The accumulation of dysfunctional mitochondria in the cell can trigger the release of mtDAMPs, which signal cellular stress and activate inflammatory pathways. Our study, focusing specifically on the liver, shows a remarkable link between hypothyroidism and activation of mtDAMPs driven-inflammation. We observed increased of mtDNA release in cytosol and enhanced protein expression of cGAS, STING, P-TBK1, P-IKBα and P65 in hypothyroid rats. All these proteins play an essential role in the mtDAMPs signalling pathways.

Upon binding to cytosolic mtDNA, cGAS produces cyclic GMP-AMP (cGAMP), which binds to and activates STING at the endoplasmic reticulum (ER) membrane. Activated STING recruits and activates TBK1, which is crucial for the downstream signalling cascade. The cGAMP-STING-TBK1 axis can activate the NF-κB signalling pathway. This involves phosphorylation and subsequent degradation of the inhibitor protein IKBα, allowing NF-κB (like the p65 subunit) to migrate into the nucleus and activate transcription of pro-inflammatory genes. We also found increased serum levels of the pro-inflammatory cytokines IL-1β, IL-6 and TNFα. Our findings indicate that hypothyroidism exacerbates liver inflammation and promotes the release of pro-inflammatory cytokines. Recent studies have highlighted the association between hypothyroidism and the immune system alterations, particularly an increase in pro-inflammatory cytokines (103–105). In addition, further research has provided insights into the involvement of the cGAS/STING signalling pathway in the context of liver inflammation, highlighting its importance in the release of inflammatory mediators and its impact on liver pathologies (106–108). This study also addresses the effects of 3,5-T2 and T3 administration to hypothyroid rats on mtDAMPs signalling pathways. Administration of T3 to hypothyroid rats appears to have a complex effect on the inflammatory response, with changes in specific protein phosphorylation and cytokine levels. Expression of liver proteins P-TBK1 and P-IKBα and serum levels of IL-1β are decreased by T3, while levels of IL-6 are increased. Although this last result may seem contradictory, it is known in the literature that hyperthyroidism, a condition comparable to the supraphysiologic T3 dose used in our experimental model, is associated with the activation of proteins related to the inflammatory response (109) through the release of pro-inflammatory cytokines into the bloodstream, including IL-6 (110, 111), which in turn promotes lipid peroxidation and free radical formation. (112–114). IL-6 is a cytokine that has numerous and diverse biological functions, and it is well-known for its pleiotropic effects (i.e. the immune system, skeletal muscle, and nervous system, hematopoietic system, liver function and cancer). Its pro- and anti-inflammatory activities are determined by its cellular sources and the phase of inflammatory processes (115). Administration of 3,5-T2 to hypothyroid rats appears to have a more pronounced anti-inflammatory effect, as shown by the decrease in cytosolic mtDNA and the expression of liver proteins associated with the cGAS/STING signalling pathway (including C-GAS, STING, P-TBK1, IKBα and P65), as well as the reduction of pro-inflammatory cytokines such as IL-1β and IL-6. In addition, 3,5-T2 increases serum levels of the anti-inflammatory cytokine IL-10. Consistent with this, we have previously shown that administration of 3,5-T2 to HFD-fed obese rats resulted in a reduction in the expression and serum levels of IL-6, IL-1β and TNFα and an increase in IL-10 (17).

Overall, we have thoroughly investigated the molecular mechanisms underlying mitochondrial liver dysfunction in hypothyroidism, identifying oxidative stress and the resulting alteration of MQC as the supposed cause of mtDAMPs. To date, there are no studies in the literature demonstrating a link between hypothyroidism and activation of mtDAMPs signalling pathways in the liver. The novelty and significance of our study emphasises the potential involvement in initiation of hepatic inflammation in hypothyroidism. Unlike T3, 3,5-T2 is able to reverse the activation of the cGAS-STING-TBK1 inflammatory axis, paving the way for the development of new strategies against inflammatory diseases associated with liver and thyroid dysfunction.

## Data Availability

The original contributions presented in the study are included in the article/**Supplementary Material**, further inquiries can be directed to the corresponding authors.
