# Supplementary material for: Comparative effects of 3,5-diiodo-L-thyronine and 3,5,3’-triiodo-L-thyronine on mitochondrial damage and cGAS/STING-driven inflammation in liver of hypothyroid rats
Source: Front Endocrinol (Lausanne). 2024 Sep 5;15:1432819. doi: 10.3389/fendo.2024.1432819 (PMC11410700; doi:10.3389/fendo.2024.1432819)
Supplement: Supplementary file 1 [file DataSheet1.doc]

Supplementary Material

# Comparative effects of 3,5-Diiodo-L-Thyronine (3,5-T2) and 3,5,3'-Triiodo-L-Thyronine (T3) on mitochondrial damage and cGAS/STING-driven inflammation in liver of hypothyroid rats

Antonia Giacco^1Ϯ^, Giuseppe Petito^2 Ϯ^, Elena Silvestri^1^, Nicla Scopigno^1^, Michela Vigliotti^1^, Giovanna Mercurio^1^, Pieter de Lange^2^, Assunta Lombardi^3^, Maria Moreno^1^, Fernando Goglia^1^, Antonia Lanni^2^, Rosalba Senese^2*^ and Federica Cioffi ^1*^

^1^Department of Science and Technologies, University of Sannio, Benevento, Italy.

^2^Department of Environmental, Biological and Pharmaceutical Sciences and Technologies, University of Campania "L. Vanvitelli", Caserta, Italy.

^3^Department of Biology, University of Naples Federico II, Napoli, Italy.

*** Correspondence:** Federica Cioffi [fecioffi@unisannio.it](about:blank)

Senese Rosalba [rosalba.senese@unicampania.it](mailto:rosalba.senese@unicampania.it)

# Figure S1.


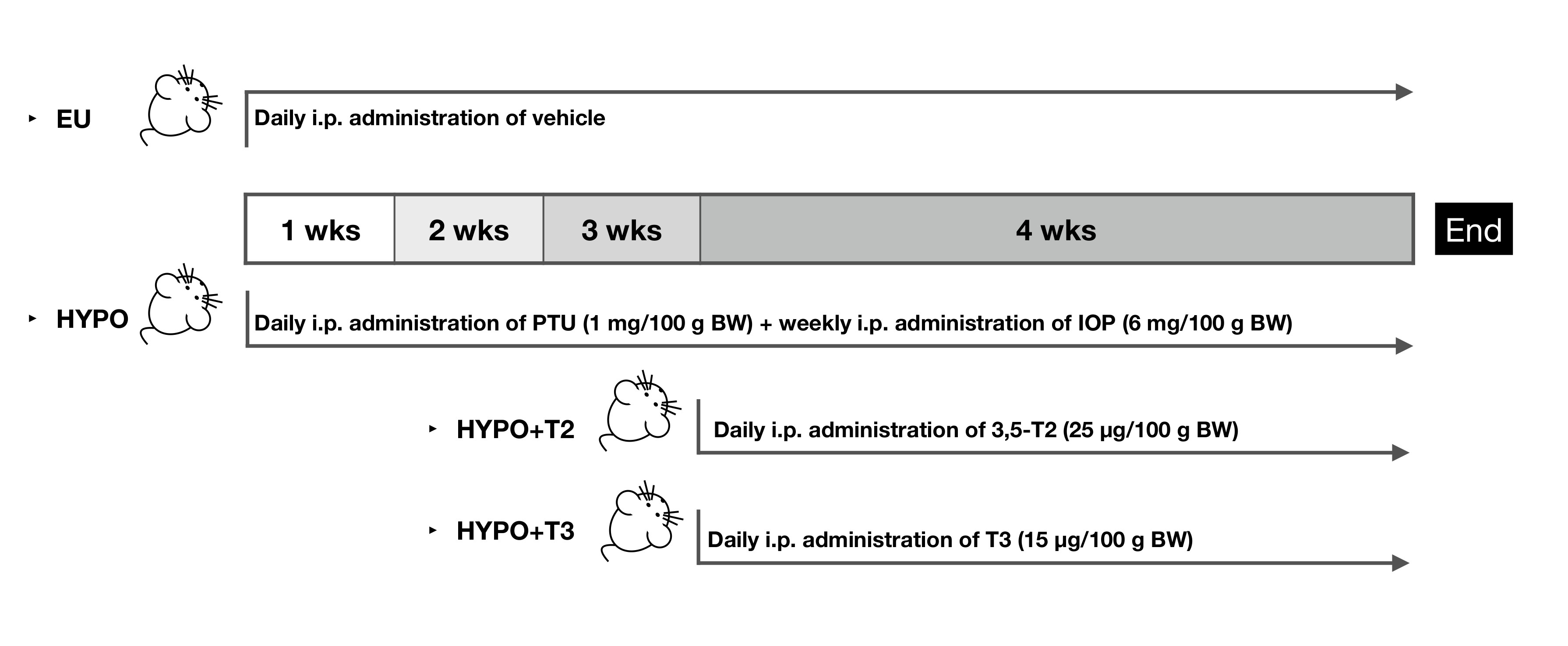


# Figure S1. Schematic representation of experimental treatment. “Eu” group consisted of euthyroid animals injected with vehicle. “Hypo” group consisted of rats made hypothyroid by daily intraperitoneal (ip) administration of propylthiouracil, PTU (1 mg/100 g body weight) together with a weekly ip injection of iopanoic acid, IOP (6 mg/100 g body weight) for 4 weeks. The “Hypo +T2” and “Hypo+T3” groups were treated like the Hypo group, but in the last week the “Hypo +T2” received an additional daily injection of 3,5-T2 at the dose of 25µg/100 g body weight, while the “Hypo+T3” received a daily injection of T3 at the dose of 15µg/100 g body weight.

# Table S1. Primers sequences.

| **Gene name** | **FORWARD** | **REVERSE** |
| --- | --- | --- |
| **ape1** | 5’-CAGATCAGAAAACGTCAGCCAG-3’ | 5’- GGTCTCTTGGAGGCACAAGA TG-3’ |
| **dio1** | 5’- GCCTTGAATGAAATCCCAGA-3’ | 5’- AGACTGGAAGACAGGGCTGA-3’ |
| **dio3** | 5’- ACAGATGAGCACAGCCACAG-3’ | 5’- CCAGAAAGCAAGCCAAAAAG-3’ |
| **mct10** | 5’- GTGCAATGGGTCTGTGTTTG-3’ | 5’- CCATGTTGTCATCGTCCTTG-3’ |
| **mct8** | 5’- ACAGCGCTTTCTGGTTCAGT-3’ | 5’- AAGGCCCAGATACGGTAGGT-3’ |
| **ogg1** | 5’ -GACTCAGACCGAGGATCAGCTC-3’ | 5’ -GCTATAGAGCTGAGTCA GGCTGAC-3’ |
| **pgc1α** | 5’ -GGAGCAATAAAGCAAAGAGCA-3’ | 5’-GTGTGAGGAGGGTCATCG TT-3’ |
| **polγ** | 5’ -CTCCTACCTGCCTGTCAACC-3’ | 5’ -GCTCCATCAGCGACTTCTTC-3’ |
| **trα** | 5’- ACCTCCATCCCACCTATTCC-3’ | 5’- CACTGATTCCGGGTGATCTT-3’ |
| **trβ** | 5’- CCCATGCAACTGAATGTACG-3’ | 5’- CCAGGCTAAGGCAGTGAAAG-3’ |

**Table S2. Antibodies.**

| **Antibody** | Reference number |
| --- | --- |
| AMBRA1 | Cell Signaling (Danvers, USA)-cod.#24907 |
| APE1 | Novus Biologicals (Centennial, USA)- cod. NB100-116 |
| ATG16L1 | Novus Biologicals (Centennial, CO, USA)-cod. NB110-82384 |
| ATG5 | Novus Biologicals (Centennial, CO, USA)-cod. NB110-53818 |
| Catalase (CAT) | Sigma (Merck, Germany)-cod CO979 |
| cGAS | HUABIO (Woburn, Massachusetts, USA)-cod. HA500023 |
| DIO1 | Proteintech (North America)-cod. 11790-1-AP |
| DIO3 | Novus Biologicals (Centennial, USA)- cod. NB05767 |
| DNA Polymerase gamma (POLΥ) | Novus Biologicals (Centennial, USA)- cod. NB110-538185 |
| DRP1 | Abcam (Cambridge, UK)- cod.ab56788 |
| Glutathione Peroxidase 1 (GPX1) | GeneTex (California, USA)-cod. gtx 03346 |
| Glutathione Peroxidase 4 (GPX4) | Abcam (Cambridge, UK)- cod.125066 |
| IKBα | Cell Signaling (Danvers, USA)- cod.#4814 |
| LC3BII | Abcam (Cambridge, UK)- cod. ab192890 |
| MCT8 | Proteintech (North America)-cod. 20676-1-AP |
| OGG1 | Novus Biologicals (Centennial, USA)-cod. NB100-106 |
| OMA1 | Abcam (Cambridge, UK)-cod. ab154949 |
| OPA1 | Abcam (Cambridge, UK)- cod.ab157457 |
| P65 | Santa Cruz Biotechnology (Dallas, TX, USA)-cod. Sc8008 |
| PARKIN | Cell Signaling (Danvers, MA, USA)-cod. #4211 |
| Peroxiredoxin 3 ( PRDX3) | Abcam (Cambridge, UK)-cod ab73349 |
| PGC1α | Abcam (Cambridge, UK)-cod.ab61249 |
| Phospho-IKBα | Cell Signaling (Danvers, USA)-cod.#2859 |
| Phospho-TBK1/NAK | Cell Signaling (Danvers, USA)-cod. 5483 |
| Phospho-ULK1(ser757) | Cell Signaling (Danvers, USA)- cod. #14202 |
| PINK1 | Abcam (Cambridge, UK)-cod. ab186303 |
| SOD2/MnSOD (SOD2) | Abcam (Cambridge, UK)- cod.ab613533 |
| SQSTM1/P62 | Cell Signaling (Danvers, MA, USA)-cod. #5114 |
| STING | Abcam (Cambridge, UK)-cod. ab227704 |
| TBK1/NAK | Cell Signaling (Danvers, USA)-cod.#3504 |
| THRβ | Santa Cruz Biotechnology (Dallas, TX, USA)-cod. sc-10822 |
| TOM20 | Cell Signaling (Danvers, MA, USA)-cod. #42406 |
| ULK1 | Cell Signaling (Danvers, USA)-cod. #8054 |
| β-ACTIN (β-ACT) | GeneTex (California, USA)-cod. gtx 109639 |

**Figure S2.**


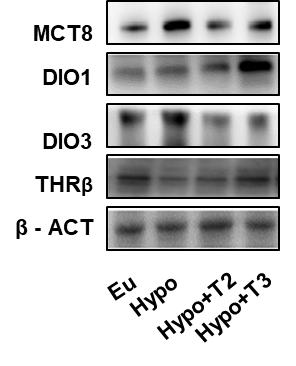

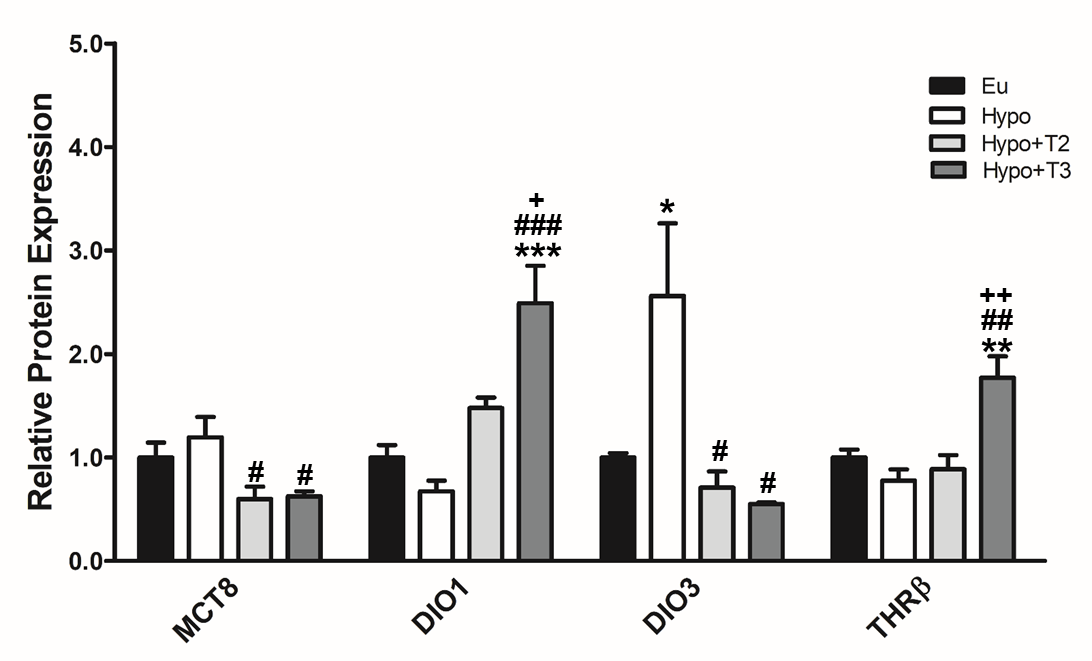


**Figure S4. Hepatic protein expression of TH transporter (MCT8), iodothyronine deiodinases (DIO1 and DIO3) and TH receptor β (THRβ) in Eu, Hypo, Hypo+T2 and Hypo+T3 groups.** Quantification of bands intensity of protein expression of MCT8, DIO1, DIO3 and THRβ and representative western blot panel. One-way Anova, Student-Newman-Keuls post-test was performed *p<0.05 vs Eu; **p<0.01 vs Eu, ***p<0.001 vs Eu; #p<0.05 vs Hypo, ##p<0.01 vs Hypo; ###p<0.001 vs Hypo; +p<0,05 vs Hypo+T2, ++p<0.01 vs Hypo+T2. For MCT8 Student’s t-test was performed #p<0.05 vs Hypo.
